# DDX5 and DDX17—multifaceted proteins in the regulation of tumorigenesis and tumor progression

**DOI:** 10.3389/fonc.2022.943032

**Published:** 2022-08-03

**Authors:** Kun Xu, Shenghui Sun, Mingjing Yan, Ju Cui, Yao Yang, Wenlin Li, Xiuqing Huang, Lin Dou, Beidong Chen, Weiqing Tang, Ming Lan, Jian Li, Tao Shen

**Affiliations:** ^1^ The Key Laboratory of Geriatrics, Beijing Institute of Geriatrics, Institute of Geriatric Medicine, Chinese Academy of Medical Sciences, Beijing Hospital/National Center of Gerontology of National Health Commission, Beijing, China; ^2^ Peking University Fifth School of Clinical Medicine, Beijing, China

**Keywords:** DDX5, DDX17, RNA transcription regulation, posttranslational modification, tumorigenesis, tumor progression

## Abstract

DEAD-box (DDX)5 and DDX17, which belong to the DEAD-box RNA helicase family, are nuclear and cytoplasmic shuttle proteins. These proteins are expressed in most tissues and cells and participate in the regulation of normal physiological functions; their abnormal expression is closely related to tumorigenesis and tumor progression. DDX5/DDX17 participate in almost all processes of RNA metabolism, such as the alternative splicing of mRNA, biogenesis of microRNAs (miRNAs) and ribosomes, degradation of mRNA, interaction with long noncoding RNAs (lncRNAs) and coregulation of transcriptional activity. Moreover, different posttranslational modifications, such as phosphorylation, acetylation, ubiquitination, and sumoylation, endow DDX5/DDX17 with different functions in tumorigenesis and tumor progression. Indeed, DDX5 and DDX17 also interact with multiple key tumor-promoting molecules and participate in tumorigenesis and tumor progression signaling pathways. When DDX5/DDX17 expression or their posttranslational modification is dysregulated, the normal cellular signaling network collapses, leading to many pathological states, including tumorigenesis and tumor development. This review mainly discusses the molecular structure features and biological functions of DDX5/DDX17 and their effects on tumorigenesis and tumor progression, as well as their potential clinical application for tumor treatment.

## 1 Introduction

The DEAD-box (DDX) protein family is the largest family of RNA helicases and consists of enzymes that unwind double-stranded RNA. DDX protein family members contain a specific D-E-A-D amino acid motif composed of aspartic acid (Asp, D)–glutamic acid (Glu, E)– alanine (Glu, A)–D (1). *DDX5* and *DDX17* share the greatest homology among the DDX gene family members. DDX5 and DDX17 are abnormally expressed in many tumors and participate in tumorigenesis, tumor cell proliferation, invasion and metastasis, which exert a profound impact on cancer development. Except for liver cancer (2) and pancreatic ductal adenocarcinoma (3), DDX5 is overexpressed in most tumors, such as breast cancer (4), prostate cancer (5), endometrial cancer (6), gastric cancer (7), esophageal cancer (8), colorectal cancer (9), glioma (10), lung adenocarcinoma (11), cervical squamous cell carcinoma (12), and non-small cell lung cancer (NSCLC) (13) etc. DDX17 is also overexpressed in many tumors, such as drug (gefitinib)-resistant NSCLC (14), prostate cancer (15), breast cancer (16), glioma (17), and hepatocellular carcinoma (18) etc. Therefore, DDX5 and DDX17 are potential targets for cancer therapy. It is necessary to explore the mechanism of action of DDX5/DDX17 in cancer to provide a solid theoretical basis for the application of DDX5/DDX17 in cancer diagnosis and treatment. The exact mechanism of DDX5/DDX17 in tumorigenesis varies with tumor type and tumor development stage. This review discusses the protein structure and biological functions of DDX5/DDX17 and explores their regulatory mechanisms in tumorigenesis and tumor progression, providing insights into their potential clinical application in tumor therapy.

## 2 Structural features of DDX5 and DDX17

*DDX5* and *DDX17* share the greatest homology among the DEAD-box gene family members; the sequence homology of their helicase core region is as high as 90%. The core region contains 9 conserved motifs shared by all DDX family members and is critical for RNA binding, ATP binding and hydrolysis functions. However, due to differences in their N-terminal and C-terminal sequences, which share 60% and 30% homology, respectively, DDX5 and DDX17 have their own unique functions in cells (19). The DDX5 and DDX17 antibodies used in the studies were designed mainly to recognize the amino acid sequence for either the N-terminal or C-terminal, which have significant differences and have no cross-reaction between the two proteins (20–24).

By querying databases such as GenBank and UniProt, we know that human *DDX5* is located on 17q23.3, while *DDX17* is located on 22q13.1, and they are expressed in all tissues. The transcription and translation of the *DDX5* gene produces a stable protein with a molecular weight of 68 kDa, also known as p68, comprising 614 amino acids. Due to alternative splicing and the alternative translation initiation codons (including a non-AUG [CUG] start codon), *DDX17* generate two stable proteins that have molecular weights of 72 kDa and 82 kDa after transcriptional and translational modifications; these proteins are also known as p72 and p82, respectively, and comprise 650 and 729 amino acids. *DDX5* and *DDX17* have been highly conserved throughout biological evolution. The genetic similarity of human *DDX5* to chimpanzee is 99.57%, to dog is 96.63, and to mice is 94.08%. The genetic similarity of human *DDX17* to chimpanzee is 99.79%, to mice is 90.15%, and to dog is 95.3%. However, the gene similarity of *DDX5* and *DDX17* to *DBP2* in yeast is only 58.94% and 55%, respectively, indicating that *DDX5/DDX17* play important roles in the life activities of higher organisms. This finding does not mean that the homologous genes of *DDX5/DDX17* are not important in lower organisms. In *S. cerevisiae*, DBP2 is involved in ribosome biogenesis, mRNA nuclear transport and degradation (25). DDX5 and DDX17 are nucleoplasmic shuttle proteins. DDX5 performs nucleocytoplasmic shuttling through the classic Ran GTPase-dependent pathway, mediated by two nuclear localization signals (NLSs) and two nuclear exporting signal (NES) sequence elements (26). DDX17 carries out nucleocytoplasmic shuttling by an exportin/importin-dependent pathway mediated by two NLSs and four NESs (14). DDX5/DDX17 perform nucleocytoplasmic shuttling, which can help other proteins carry out nucleocytoplasmic transport. For example, DDX5/DDX17 mediate the nuclear transport of β-catenin and promote the translocation of β-catenin from the cytoplasm to the nucleus, inducing tumorigenesis and tumor progression (14, 27).

## 3 Involvement of DDX5/DDX17 in RNA metabolism and cancer development

As unitary members of the RNA helicase family, DDX5/DDX17 exhibit RNA-dependent ATP hydrolase activity and RNA helicase activity. DDX5/DDX17 also show important RNA annealing activity, most notably in catalyzing the rearrangement of the RNA secondary structure in conjunction with their RNA helicase activity (28). DDX5/DDX17 are involved in almost all processes of RNA metabolism, including RNA selective splicing, biogenesis of miRNA and ribosomes, R-loop resolution, interaction with long noncoding RNAs, and regulation of transcriptional activity as a transcription factor.

### 3.1 Adjustments to hnRNA splicing/alternative splicing

After initial transcription, the precursor mRNA (heterogeneous nuclear RNA, hnRNA) is spliced to generate a mature mRNA. Only mature mRNA can enter the cytoplasm and be translated into proteins. Recently, many studies have shown that alternative splicing closely correlates with the expression of many cancer-promoting genes and tumorigenesis. Moreover, cancer-specific splice variants may be used as diagnostic biomarkers for cancer (29).

DDX5 and DDX17 play critical roles in hnRNA alternative splicing. DDX17 may facilitate U1 snRNP recognition of and binding to the 5’ splice site of mRNA (30), while DDX5 is critical for the excision of U1 snRNP from the 5’ splice site and plays an important role in the prospliceosome transition into the spliceosome (31). In myoblasts and epithelial cells, DDX5/DDX17 facilitate the binding of hnRNP H/F to G-tracts, enhancing their splicing function, and coregulate the splicing of specific exon subsets with hnRNP H/F. Downregulation of DDX5/DDX17 expression during myogenesis and the epithelial-mesenchymal transition (EMT) regulates cell-specific alternative splicing and phenotypic changes, leading to changes, for example, in the formation of lamellipodia (32). DDX5 is involved in the selective splicing of H-Ras proto-oncogenes. H-Ras can be selectively spliced into two proteins, p21 H-Ras and p19 H-Ras. DDX5 produces the oncogene p21 H-Ras by inhibiting the recognition and splicing of the intron D exon (IDX), which can contribute to the development of cancer (33). DDX17 is also involved in the splicing of CD44, which is an extremely broad cell surface transmembrane glycoprotein and is mainly involved in the adhesion of tumor cells to host cells and the host matrix. Aberrant alternative splicing of CD44 is closely related to tumorigenesis and tumor progression. Ameur et al. found that under NF-κB activation, the NF-κB family member RELA bound to the vicinity of genomic exons and recruited DDX17 to regulate splicing *via* its RNA helicase activity, increasing the inclusion rate of alternative exons v10 in the CD44 gene (34). In addition, Honig et al. found that an increase in DDX17 expression *in vivo* can lead to an increased rate of alternative exon v4 and v5 inclusion in the human CD44 gene (35). CD44 gene expression is regulated by the androgen receptor (AR), which can inhibit the splicing of variable exons in the CD44 gene. DDX5 does not directly interact with CD44 but regulates CD44 splicing by acting on the AR (5). In hepatocellular carcinoma cells, DDX17 is overexpressed, and DDX17 induces intron 3 retention in PXN-AS1, promoting the generation of the PXN-AS1-IR3 transcript. PXN-AS1-IR3 recruits Tex10 and p300 to the MYC enhancer region to promote MYC transcriptional activation, thereby promoting the migration and invasion of hepatocellular carcinoma (36). In addition, DDX5 and/or DDX17 are also involved in the alternative splicing of Caspase 9, mH2A1 (37), macroH2A1 (38), etc., which increased the malignancy of pancreatic ductal adenocarcinoma and breast cancer.

**Figure 1:**
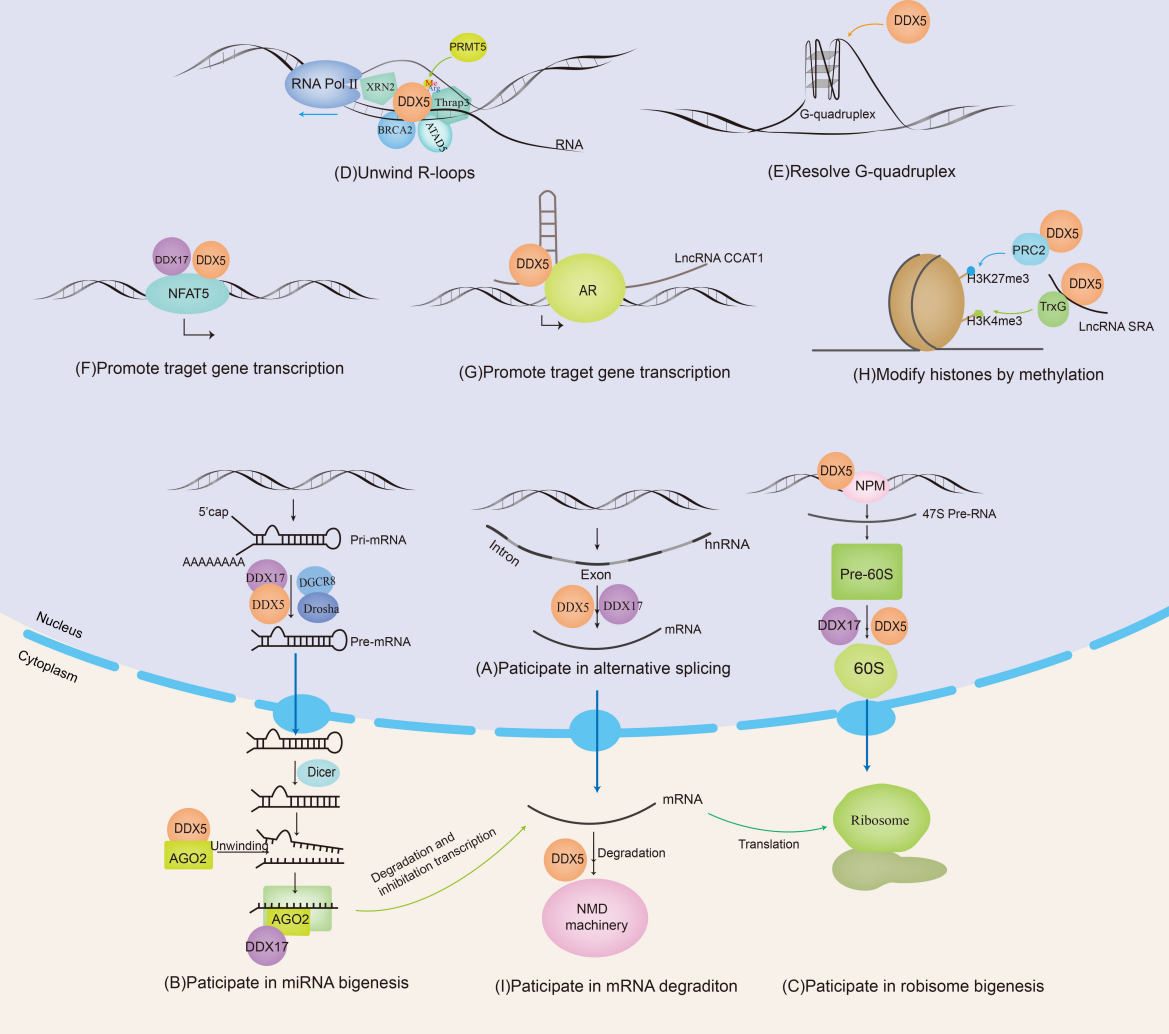
DDX5 and DDX17 are involved in RNA metabolism. DDX5/DDX17 are involved in ribosome synthesis, microRNA (miRNA) biosynthesis, regulation of mRNA alternative splicing, and the promotion or inhibition of mRNA transcription. DDX5 is also involved in nonsense-mediated mRNA decay (NMD), unwinding of R-loops and G-quadruplexes.

Together, DDX5/DDX17 regulate various physiological and/or pathological functions by modulating the alternative splicing of specific genes. For instance, H-Ras, CD44, PXN-AS1, macroH2A1, etc., are important tumor-related factors; therefore, the dysregulation of DDX5/DDX17 action is closely related to tumorigenesis and tumor progression (**Figure 1A**).

### 3.2 Participation in miRNA biogenesis

MiRNAs are noncoding RNAs approximately 18-24 nucleotides long that regulate gene expression by silencing targeted mRNAs. Primary miRNAs (pri-miRNAs) are transcribed by RNA polymerase II or RNA polymerase III and undergo two major maturation steps catalyzed by enzymes in the RNase III family. In the first step, pri-miRNAs are spliced into precursor miRNAs (pre-miRNAs) by DROSHA/DGCR8 in the nucleus, and these pre-miRNAs are exported to the cytoplasm by exportin 5 transporters. In the second step, pre-miRNAs are cleaved by Dicer in the cytoplasm to form a mature double-stranded miRNA. After unwinding, one of the miRNA strands and other proteins are selectively loaded onto Ago to form RNA-induced silencing complexes (RISCs), recognizing sites in the 3’ UTR of target mRNAs and inhibiting protein expression (39–41).

DDX5/DDX17 are positive regulators of the DROSHA/DGCR8 basic microprocessor complex. Other positive regulators include SMADs, p53, HNPNP-AI, KSPR, SF2/ASF, SNIP1, BRCA1, etc. Negative regulatory factors include YAP, MeCP2, NF90-NF45, Lin28, ADAR1/2, etc. (42). A recent crystallography experiment showed that the core catalytic domains of DDX17 had an RMFQ motif, which is important for RNA recognition, enhancing pri-miRNA processing through the VCAUCH motif in the pri-miRNA 3’ flanking region (43). DDX5/17 selectively recruits pri-miRNAs and efficiently cleaves pri-miRNA at specific sites through DROSHA, such as miR-21 (44), miR-132 (45), and miR-125b (46). DDX5 can also unwind the pre-miRNA double chain in the cytoplasm and load a single chain into the silencing complex, such as miRNA let-7 (47). In addition, it has been reported that the loss of DDX17 and KHSRP (KH-type splicing regulatory protein) can cause a decrease in the Ago protein level, and both DDX17 and KHSRP can modulate the stability of Ago2 by regulating the intracellular miRNA level (48) (**Figure 1B**). In conclusion, DDX5/DDX17 play an important role in two key steps of miRNA maturation. DDX5/DDX17 can regulate the stability of specific mRNAs by regulating miRNA biosynthesis, thus modulating protein expression and cellular biological functions. Notably, miRNAs such as miR-21 (49), miR-132 (50), miR-125b (51), and let-7 (52) mentioned above are dysregulated in various cancers; thus, when DDX5/DDX17 are abnormally expressed, the cellular information network is dysregulated, providing opportunities for tumor cell invasion.

### 3.3 Participation in ribosome biogenesis

The ribosome is the site of protein synthesis and is composed mainly of ribosomal RNA (rRNA, which is produced through rDNA transcription) and proteins. Assembly of the ribosome begins at nucleoli, forming a pre-90S ribosome from nonribosomal protein, ribosomal protein, and a single pre-rRNA primary transcript (47S in mammals). The primary pre-rRNA transcript undergoes rapid cleavage, and the 90S pre-ribosome is divided into large and small subunit precursors. In the large subunit precursor (60S pre-ribosomal particle), pre-rRNA matures into 28S rRNA and 5.8S rRNA. Early 60S pre-ribosomes are confined to the nucleolus, and the mature 60S ribosome subunit is subsequently released into the nucleus and ultimately exported to the cytoplasm (53–55).

With the participation of U8 small nucleolar RNA (SnoRNA), DDX5 and DDX17 may promote the rearrangement of RNA structures within the 60S pre-ribosomal subunit, which is essential for the proper temporal cleavage of the pre-28S rRNA (22). In addition to their interaction with U8SnoRNA, DDX5/DDX17 also participate in the regulation of U3snoRNA and U13snoRNA (24). Research data have shown that, except at the end of mitosis (22), most endogenous DDX5 proteins are excluded from nucleoli and therefore are inactive during ribosomal biogenesis until signaling stimulates DDX5 activity. ARF contributes to the nucleolar sequestration of DDX5, inhibits DDX5 interaction with nucleophosmin (NPM), and prevents DDX5 association with rDNA promoters and pre-ribosomes. However, in most cancers, ARF expression is significantly decreased, then a significant increase in nucleolar DDX5 expression has been observed. NPM promotes the binding of DDX5 to the rDNA promoter and promotes 47S pre-rRNA transcription (56, 57). DDX5 stimulates ribosome biogenesis and increases protein production, consistent with the higher metabolic rate required by cancer cells during tumorigenesis. DDX5 may represent a class of non-oncogenes with activity that is stimulated in the absence of key tumor suppressors (**Figure 1C**).

### 3.4 Participation in R-loop resolution

R-loops are instantaneously and reversibly formed DNA/RNA hybrids and displaced single-stranded DNA. R-loops are involved in many physiological processes, such as transcription and class switch recombination. The formation of abnormal transcription-related R-loops can cause catastrophic replication dysregulation, leading to DNA double strand breaks (DSBs) and genomic instability (58). Wells et al. found that when the proto-oncogene was activated or the tumor suppressor gene was suppressed, R-loops and the associated DNA damage were increased significantly (59).

DDX5 is involved in the R-loop resolution. Genome-wide DNA/RNA immunoprecipitation sequencing revealed that in DDX5-deficient cells, the accumulation of R-loops near transcription start sites, transcription stop sites, and DSB regions was increased. DDX5 deletion delayed the recruitment of exonuclide 1 and replication protein A to laser-induced DNA damage sites, resulting in homologous recombination repair defects. Therefore, DDX5 promotes the clearance of abnormal RNA transcripts from DSB sequences to ensure appropriate DNA repair (60). The RGG/RG motif in the C-terminus of DDX5 can be methylated by protein arginine methyltransferase 5 (PRMT5). Methylated DDX5 recruits exonuclease XRN2 and degrades R-loops in the transcription termination region downstream of the poly(A) site to promote the release of RNA polymerase II, thus promoting smooth transcription (61). In this process, thyroid hormone receptor-associated protein 3 (Thrap3) binds to methylated DDX5 to promote the recruitment of XRN2 to R-loops (62). BRCA2 is a DSB repair factor. The combination of BRCA2 and DDX5 can also promote DDX5 unwinding of DNA–RNA hybrid complexes (63). ATAD5 increases the abundance of DDX5 at replication forks and promotes R-loop resolution (64). Sox2 interacts with DDX5 and inhibits DDX5 from resolving the R-loops, thereby promoting reprogramming (65). Therefore, DDX5 contributes to promoting smooth transcription and ensuring the stability of the genome by its resolvase activity on R-loops. When tumor suppressor genes are inactivated or proto-oncogenes are activated, the formation of R-loops is significantly increased. However, it is unclear whether the resolvase activity of DDX5 on R-loops exerts oncogenic or tumor suppressive effects; this question will be an interesting topic for further investigation (**Figure 1D**).

### 3.5 DDX5/DDX17 are gene transcription regulators

DDX5 and DDX17 are both important transcriptional regulators and act as either coactivators or cosuppressors depending on the gene promoter and the transcriptional complex in which they are located.

DDX5/DDX17 can be coactivators in gene transcription. *MYC* is an oncogene that plays an important role in the malignant progression of many human tumors. A G-quadruplex (G4) is a higher-order structure formed by the folding of DNA or RNA rich in guanine tandem repeats. When the oncogene *MYC* is not actively transcribed, the inherent superhelix does not generally form G4 in the *MYC* double-stranded promoter region. However, in highly transcribed cells, metastable G4 structures can spontaneously form. In these cells, G4 acts as a transcriptional silencing element by preventing transcription factor binding to the *MYC* promoter. However, DDX5 is a highly active G4-resolvase that can effectively unfold the G4 structure and thus promote the transcription of the *MYC* gene to promote tumorigenesis and tumor progression (66) (**Figure 1E**). NFAT5 is involved in the cellular response to osmotic pressure and in the migration of breast cancer cells. DDX5/DDX17 increase the binding efficiency of NFAT5 to its target genes, which promotes the expression of migration-related genes (20) (**Figure 1F**). See **Table 1** for more details.

**Table 1:** DDX5/DDX17 coactivate or corepress transcription factor transcription.

DDX5/DDX17	Coactivation (↑)/Corepression(↓)	Transcription factor	Physiological or pathological function	References
DDX5/DDX17	↑	SMAD	Initiate EMT and myogenesis in the differentiation of epithelial cells and myoblasts	(32)
DDX5/DDX17	↑	NFAT5	Enhance the transcription of NFAT5 target genes, but negatively regulate NFAT5 mRNA, then involve in precise regulation of breast cancer cell migration.	(20)
DDX5/DDX17	↑	MyoD	Promote the transformation of fibroblasts in skeletal muscle cells.	(67)
DDX5/DDX17	↑	ERα	Play important roles in the development of breast cancer.	(16, 19, 68)
DDX5/DDX17	↑	p53	Involved in cell cycle arrest and DNA damage.	(69–71)
DDX17	↑	Sox2	Increase the stemness of estrogen receptor-positive breast cancer cells.	(16)
DDX5	↑	Runx2	Control osteoblast specification and maturation.	(72)
DDX5	↑	AR	Play important roles in the development of prostate cancer.	(5, 73)
DDX5	↑	TFEB	Spermatogenesis.	(74)
DDX5	↑	PLZF	Spermatogenesis.	(74)
DDX5	↑	VDR	Stimulate the transcription of vitamin D receptor target genes.	(75)
DDX5	↑	β-Catenin	Participate in Wnt/β-catenin signaling pathway and promote tumorigenesis.	(27)
DDX5	↑	NF-κB p50	Participate in the NF-κB signaling pathway and promote tumorigenesis.	(76)
DDX5	↑	Stat3	Participate in the Stat3 signaling pathway and promote colon cancer progression.	(77)
DDX5	↑	NOTCH1/RBP-J	Participate in the NOTCH signaling pathway and promote tumorigenesis.	(78)
DDX5	↑	Fra-1	Promote Fra1-mediated cell proliferation and promote the progression of triple-negative breast cancer.	(79)
DDX5	↑	E2F	Directly regulate the expression of DNA replication factors.	(80)
DDX5	↑	IL-1β	Promote glioma proliferation and neutrophil recruitment.	(81)
DDX5	↑	Fabp1	Involved in the formation of intestinal tumors and inflammation.	(82)
DDX5/DD17	↓	REST	Inhibit the transcription of REST target genes and negatively regulate the REST complex, participating in neuronal differentiation.	(83)
DDX5	↓	Pkd1	DDX5 and p53 corepress Pkd1 transcription, causing renal cyst progression and fibrosis in autosomal dominant polycystic kidney disease.	(84)
DDX17	↓	klf4	Promote the progression of hepatocellular carcinoma.	(18)

DDX5/DDX17 can be cosuppressors in gene transcription. Repressor element 1-silencing transcription factor (REST) inhibits the expression of many neuronal genes in nonneuronal cells or undifferentiated neural progenitor cells. DDX5/DDX17 promote REST binding to the promoters of a subset of REST target genes and thus coregulate REST inhibition of transcriptional activity. In the absence of DDX5/DDX17, REST binding and its cofactor EHMT2 are reduced, suggesting that DDX5/DDX17 stabilize the association of the entire REST complex with its target promoter (83). In hepatocellular carcinoma cells (HCC), DDX17 binds to the zinc finger domain of Klf4, promoting the dissociation of Klf4 from chromatin and blocking the activation of its target gene promoters, thereby modulating the target genes of Klf4, such as decreasing the expression of E-cadherin and increasing the expression of MMP-2. Thus, DDX17 can promote HCC cell migration and invasion, which lead to the progression of hepatocellular carcinoma (18). See **Table 1** for more details.

In summary, DDX5 and DDX17 play either coactivator or cosuppressor roles in cells. On the one hand, DDX5/DDX17 change the conformation of the main activator/suppressor, promoting the binding of the main activator/suppressor to a chromatin promoter or promoting the dissociation of the main activator/suppressor from a chromatin promoter. On the other hand, DDX5/DDX17 can be adaptor proteins that recruit transcription factors and thus promote transcription. For example, DDX5/DDX17 can recruit SRA, CBP/P300, P/CAF and RNA Pol II, promoting transcription coactivation complex formation (85, 86).

### 3.6 Regulation of DDX5/DDX17 by lncRNAs

Long noncoding RNAs (lncRNAs) are key epigenetic regulators of gene expression that exert an enhancing or silencing effect on promoter activity through a variety of mechanisms (87). DDX5 and DDX17 interact with multiple lncRNAs to influence chromatin status and regulate target gene transcription.

LncRNAs can “sponge” miRNAs. For example, PWRN2 (88), TINCR (89), Linc00473 (90), DLEU1 (2), MIAT (91), LINC01207 (92) FGD5-AS1 (93), SNHG14 (94) and MSC-AS1 (95) absorb the inhibitory miRNA targeting DDX5 mRNA, a process called “sponging”, thus upregulating DDX5 protein expression. Moreover, lncRNAs, as scaffold elements, can work with RNA helicases to correctly locate transcription factors and related proteins on their target gene promoters, allowing modification of local chromatin. For example, lncRNA CCAT1 acts as the scaffold of DDX5 and androgen receptor transcription complex to promote the expression of androgen receptor-targeted genes, which leads to a poor prognosis for patients with castration-resistant prostate cancer (73) (**Figure 1G**). In addition, lncRNAs can also regulate the ubiquitin–proteasome pathway of DDX5. For example, NHEG1 (96) inhibits ubiquitin–proteasome pathway-induced DDX5 degradation, while SLC26A4-AS1 (97) and PSCA (98) promote DDX5 degradation through the ubiquitin–proteasome pathway. In addition, in mouse spermatogonial cells, downregulating Mrhl expression promoted cytoplasmic translocation of tyrosine-phosphorylated DDX5, thereby promoting Wnt/β-catenin signaling pathway activation (99). In turn, DDX5 can also affect lncRNA activity; for example, DDX5 promoted selective interaction between lncRNA SRA and the TrxG complex to catalyze trimethylation of histone H3K4 in pluripotent stem cells, thereby remodeling chromatin and promoting gene transcription (100) (**Figure 1H**). Thus, lncRNAs are involved in the progression of various cancers by regulating the stability of DDX5 or DDX17 at the RNA level and at the protein level or by acting as scaffolding elements in the complex. See **Table 2** for more detailed information. This finding reinforces the critical role of DDX5 or DDX17 in carcinogenesis.

**Table 2:** Regulation of DDX5/DDX17 by lncRNAs.

lncRNA	DDX5/DDX17	Function	References
SRA	DDX5/DDX17	SRA exerts a partial synergistic effect with DDX5/DDX17, such as interactions with ERα, AR, Notch1 and MyoD; And DDX5 promotes SRA to selectively interact with TrxG complex.	(67, 78, 101)
MeXis	DDX17	MeXis combines with Abca1 promoter to increase Abca1 expression and cholesterol efflux in macrophages.	(102)
CCAT1	DDX5	CCAT1, as a scaffold linking DDX5 and AR transcription complex, promotes the development of castration-resistant prostate cancer.	(73)
NEAT1	DDX5	NEAT1 indirectly activates the Wnt/β-catenin signaling pathway through DDX5 to promotes colorectal cancer progression.	(103)
Mrhl	DDX5	In mouse spermatogonia, the downregulation of Mrhl promotes the cytoplasmic translocation of tyrosine-phosphorylated DDX5 and promotes the Wnt/β-catenin signaling pathway.	(99)
LOC284454	DDX5	LOC284454 affects the overexpression of the micro RNAs miR-23a, miR-27a and miR-24-2.	(104)
NHEG1	DDX5	NHEG1 inhibits the degradation of DDX5 through the ubiquitin–proteasome pathway to promote neuroblastoma progression.	(90)
SLC26A4-AS1	DDX5	SLC26A4 promotes the degradation of DDX5 through the ubiquitin–proteasome pathway to inhibit thyroid cancer metastasis.	(97)
LINC01116	DDX5	LINC01116 recruits DDX5 to the IL-1β promoter and activates IL-1β expression to promote neutrophil recruitment and glioma proliferation.	(81)
PWRN2	DDX5	PWRN2 sponges miR-325, to prevent miR-325 from targeting DDX5, thus promoting the progression of papillary thyroid carcinoma.	(88)
TINCR	DDX5	TINCR sponges miR-218-5p to prevent miR-218-5p from targeting DDX5, thus promoting the progression of liver cancer.	(89)
Linc00473	DDX5	Linc00473 sponges miR-506, preventing miR-506 from targeting DDX5, thus promoting the progression of cholangiocarcinoma.	(90)
DLEU1	DDX5	DLEU1 sponges miR-671-5p, making it impossible for miR-671-5p to target DDX5, thus promoting the progression of osteosarcoma.	(2)
MIAT	DDX5	MIAT sponges miR-141, making it impossible for miR-141 to target DDX5, thus promoting the progression of gastric cancer.	(91)
RMRP	DDX5	Promote the development of treatment-naïve relapsing-remitting multiple sclerosis.	(105)
LINC01207	DDX5	LINC01207 sponges miR-671-5p, inhibiting miR-671-5p targeting of DDX5 and promoting the development of gastric cancer.	(92)
FGD5-AS1	DDX5	FGD5-AS1 sponges miR-493-5p, rendering miR-493-5p incapable of targeting DDX5 and promoting the development of non-small cell lung cancer.	(93)
PSCA	DDX5	PSCA interacts with DDX5 and promotes DDX5 degradation through ubiquitination to inhibit the progression of gastric cancer.	(98)
SNHG14	DDX5	SNHG14 sponges miR-519b-3p, rendering it unable to target DDX5 for degradation and promoting colorectal cell proliferation and invasion.	(94)
MSC-AS1	DDX5	MSC-AS1 sponges miR-142-5p, rendering it unable to target DDX5 for degradation and promoting the development of gastric carcinoma.	(95)
PRADX	DDX5	PRADX recruits the PRC2/DDX5 complex to promote glioblastoma and colon cancer.	(106)
SUNO1	DDX5	SUNO1 affects DDX5 to regulate the recruitment of RNA polymerase II to a the WTIP cis promoter, enhancing the transcription of WTIP, and promoting the development of colon cancer.	(107)
Platr22	DDX5	LncRNA Platr22 binds to DDX5 to promote superenhancer activity and stem cell pluripotency.	(108)
CPhar	DDX17	CPhar regulates exercise-induced cardioprotection.	(109, 110)
MeXis	DDX17	MeXis interacts with DDX17 to facilitate cholesterol efflux in macrophages.	(102)
SNHG20	DDX17	SNHG20, as a competing endogenous RNA, upregulates DDX17 expression to promote the development of prostate cancer.	(15)

### 3.7 Participation in nonsense-mediated mRNA decay

Nonsense-mediated mRNA decay (NMD) is a posttranscriptional monitoring mechanism of mRNA that degrades mRNA containing a premature translation termination codon (PTC) and prevents the generation of diseases. Upf family members are a group of protein and are core factors involved in the formation of NMD. DDX5/DDX17 interact with Upf3 to bind to the Upf complex, activating the NMD signaling pathway (111). NFAT5 is involved in the migration of breast cancer cells. As mentioned above, DDX5/DDX17 promote the transcription of NFAT5 target genes. In addition, DDX5/DDX17 increase the inclusion of NFAT5 exon 5 at the splicing level. Because exon 5 contains a PTC, NFAT5 mRNA is degraded through the NMD pathway, leading to a decrease in NFAT5 protein levels (20) (**Figure 1I**). Therefore, DDX5/DDX17 play a key role in tumor cell migration through the fine regulation of the NFAT5 pathway.

In summary, RNA metabolism is the bridge connecting genetic genes to functional proteins. Abnormal RNA metabolism is closely related to cancer cell survival, proliferation, self-renewal, differentiation, stress adaptation, invasion and resistance to therapy. DDX5 and DDX17 are involved in almost the entire RNA metabolic process, from RNA transcription, splicing, and translation to final degradation. Thus, the important roles of DDX5/DDX17 in the body are self-evident and may be potential targets in cancer treatment.

## 4 Posttranslational modifications of DDX5 and DDX17 in cancer

Posttranslational modification refers to the chemical modification of a protein after translation that endows the modified protein with specific biological functions. Dysregulated posttranslational modifications are closely related to tumorigenesis and tumor progression. DDX5 and/or DDX17 can be phosphorylated, acetylated, ubiquitinated, sumoylated, O-GlcNAcylated, etc. Different posttranslational modifications of DDX5/DDX17 and identical posttranslational modifications at different sites play different roles in tumorigenesis and tumor progression.

### 4.1 Phosphorylation of DDX5

The T69, T446, T564, S557, Y593, and Y595 sites of DDX5 can be phosphorylated. T69-phosphorylated DDX5 by PAK5 increases binding to the Drosha/DGCR8 complex to facilitate miR-10b production, promoting breast cancer cell proliferation and migration (112). S557-phosphorylated DDX5 is a microtubule motor that binds calmodulin (CaM) to position CaM at the front of metastatic cells and thus promotes cell metastasis (113). Y593-phosphorylated DDX5 enhances the coactivation of androgen receptor transcription (5). Moreover, Y593 phosphorylation of DDX5 promotes the dissociation of histone deacetylase 1 (HDAC1) from the Snail1 promoter and thus activates Snail1 transcription, inhibiting transcription of the adhesion factor E-cadherin and promoting EMT (114). In addition, Y593-phosphorylated DDX5 promotes the nuclear translocation of β-catenin by blocking the β-catenin phosphorylation induced by GSK-3β and replacing Axin in the β-catenin complex, then activating β-catenin target genes *cyclin D1* and *c-Myc* transcription and stimulating cell proliferation (115) In glioblastoma, dual Y593-/Y595- phosphorylated DDX5 inhibits cell apoptosis by inhibiting XAF1 expression. Therefore, phosphorylation of Y593/Y595 on DDX5 promotes cell proliferation, invasion and anti-apoptosis, which are characteristics of a carcinogenic phenotype. However, the dual phosphorylation of T564/T446 on DDX5 mediated by p38 MAP kinase promotes colon cancer cells apoptosis during chemotherapeutic drug treatment (oxaliplatin) (116). These findings illustrate the complexity of DDX5 phosphorylation regulation. In some cases, the phosphorylation of different residues in a protein may exert opposite effects.

### 4.2 Acetylation of DDX5 and DDX17

DDX5/DDX17 are acetylated on several lysine residues in the N-terminal region (at K32, K33, K40, K43, K44, and K45 in DDX5 and at K29, K30, and K42 in DDX17). Acetylation enhances DDX5/DDX17 coactivation of estrogen receptor target gene transcription and it also enhances DDX17, but not DDX5, coactivation of the murine double minute 2 (MDM2) promoter in p53-dependent transcription. Increased MDM2 transcription can reduce p53 levels by establishing a p53-MDM2 negative feedback loop, promoting the malignant transformation of breast cancer. Therefore, acetylation of DDX5/DDX17 promotes their transcriptional coactivation ability. However, acetylation also promotes the DDX5/DDX17 interaction with histone deacetylases (HDACs). Specifically, acetylation enhances the DDX17 interaction with HDAC1 and HDAC3; acetylation enhances the DDX5 interaction with HDAC1 and HDAC2 (117). Therefore, acetylated DDX5/DDX17 recruited to the chromosome binds to HDAC to repress gene transcription to some extent, indicating that there may be a negative feedback mechanism mitigating overactivation of a promoter targeted by acetylated DDX5/DDX17.

### 4.3 Ubiquitination of DDX5 and DDX17

DDX5 is degraded through the ubiquitin–proteasome pathway. The lncRNA NHEG1 (96) can interact with and inhibit DDX5 degradation by the ubiquitination-triggered proteasome, while the lncRNAs SLC26A4-AS1 (97) and PSCA (98) promote DDX5 degradation through the ubiquitin–proteasome pathway. The K190 site of DDX17 can be ubiquitinated. Specifically, under hypoxic conditions, the K190 site of DDX17 undergoes K63 ubiquitination by the E3 ligase HectH9. Ubiquitinylated DDX17 dissociates from the pri-miRNA-Drosha-DCGR8 complex, reducing the biogenesis of anti-stemness miRNAs. Meanwhile, dissociated DDX17 forms the DDX17/YAP/p300 complex, which enhances the transcription of tumor stem genes. DDX5 does not have a site corresponding to K190 in DDX17 and cannot be ubiquitinated by HectH9 (118). The coordinated regulation of miRNA biogenesis and histone modification by DDX17 ubiquitination is the basis of many cancer stem cell-like characteristics.

### 4.4 Sumoylation of DDX5 and DDX17

DDX5 undergoes sumoylation at K53, and DDX17 undergoes sumoylation at K50. DDX5 is preferentially modified by SUMO-2, rarely modified by SUMO-3, and negligibly modified by SUMO-1. These findings indicate that DDX5, a sumoylation substrate, has high selectivity for sumoylation enzymes. The SUMO E3 ligase PIAS1 interacts with DDX5 and enhances DDX5 sumoylation, enhancing the inhibitory transcriptional activity of DDX5, which leads to the inhibition of both p53 activation and thymidine kinase promoter transcription. These findings may be explained by sumoylated DDX5 altering the chromatin modification state by recruiting HDAC1 (119). In contrast, another study found that sumoylation enhanced DDX5 coactivation of estrogen receptor α (ERα) but had no effect on the DDX5 coactivation of p53 (23). However, the same study (23) found that sumoylation inhibited DDX17 coactivation of ER and p53. These opposing results and conclusions may be explained by differences in cell type and pathological condition. In addition, sumoylation increases the stability of DDX5 and DDX17, notably extending the half-life of DDX5 by preventing its degradation by the proteasome. In breast cancer, sumoylation is overactivated and increases the stability of DDX5 and DDX17, which may explain the high DDX5/DDX17 expression in breast cancer (23).

### 4.5 O-GlcNAcylation and methylation of DDX5

DDX5 can be modified by O-GlcNAcylation and methylation. In the SW480 cell line (a colorectal adenocarcinoma cell line), DDX5 interacts directly with O-linked N-acetylglucosamine transferase (OGT), which is the only enzyme known to catalyze O-GlcNAcylation. OGT-mediated O-GlcNAcylation stabilizes DDX5, activates the AKT/mTOR signaling pathway, and then accelerates the progression of colorectal cancer (120). Protein arginine methyltransferase 5 methylates the RGG/RG motif in the C-terminal of DDX5. Methylated DDX5 recruits the exonuclease XRN2, which detaches and degrades the R-loop at the transcriptional termination region downstream of the poly(A) site, thus facilitating smooth transcription (61).

**Figure 2:**
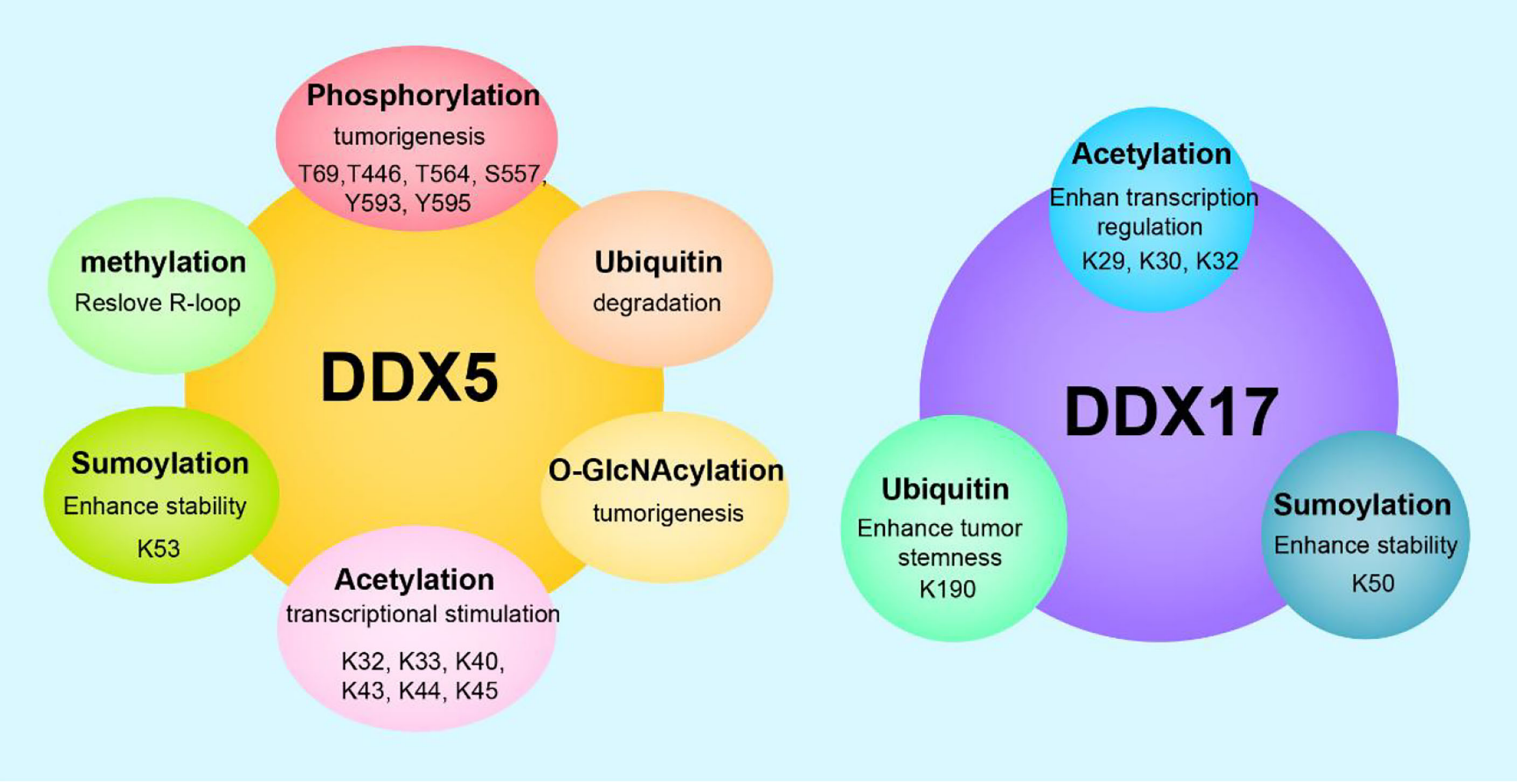
Posttranslational modifications and functions of DDX5/DDX17. DDX5 can undergo phosphorylation, acetylation, methylation, ubiquitination, sumoylation, O-GlcNAcylation, etc. DDX17 can undergo acetylation, ubiquitination, sumoylaton, etc. Different posttranslational modifications endow DDX5/DDX17 with diverse biological functions, and the same modification at different sites leads to different functional outcomes.

In conclusion, in most cases, the phosphorylation of DDX5 promotes cell proliferation and invasion; the acetylation of DDX5/DDX17 promotes their transcriptional coactivation ability; K48 ubiquitination regulates DDX5 degradation through the ubiquitin–proteasome pathway; K63 ubiquitination on DDX17 coordinates the regulation of miRNA biogenesis and histone modification, increasing the stemness of cancer stem cells; sumoylation increases the stability of DDX5/DDX17 and enhances the inhibitory transcriptional activity of DDX5/DDX17; O-GlcNAcylation of DDX5 promotes tumor development.; and methylated DDX5 is involved in R-loop resolution. Therefore, the posttranslational modification of DDX5/DDX17 is closely related to tumorigenesis and tumor progression, and in-depth research on the mechanisms of these actions will provide new ways to explore tumor pathogenesis and develop tumor therapies (**Figure 2**).

## 5 DDX5 and DDX17 regulate major cancer signaling pathways

Various signaling molecules are connected, interacting with each other and restricting each other to form a signaling network system. DDX5/DDX17 interact with many key tumor signaling molecules and participate in a variety of tumor-regulated signaling pathways, such as DNA damage repair, autophagy, oxidative stress and energy metabolism. Therefore, once DDX5/DDX17 expression is dysregulated, the cell signaling pathway will be disturbed, leading to tumorigenesis and tumor progression.

### 5.1 The interaction of DDX5/DDX17 with key signaling molecules in tumors

In the signaling pathway network, the dysregulation of the key molecules can drive the entire system to deviate off course and can cause malfunctions in which normal physiological functions are lost, resulting in tumorigenesis and tumor development. Important tumor-related signaling molecules regulated by DDX5/DDX17 include p53, Wnt/β-catenin, Notch, estrogen and androgen, YAP, and NF-κB.

#### 5.1.1 DDX5/DDX17 and p53

**Figure 3:**
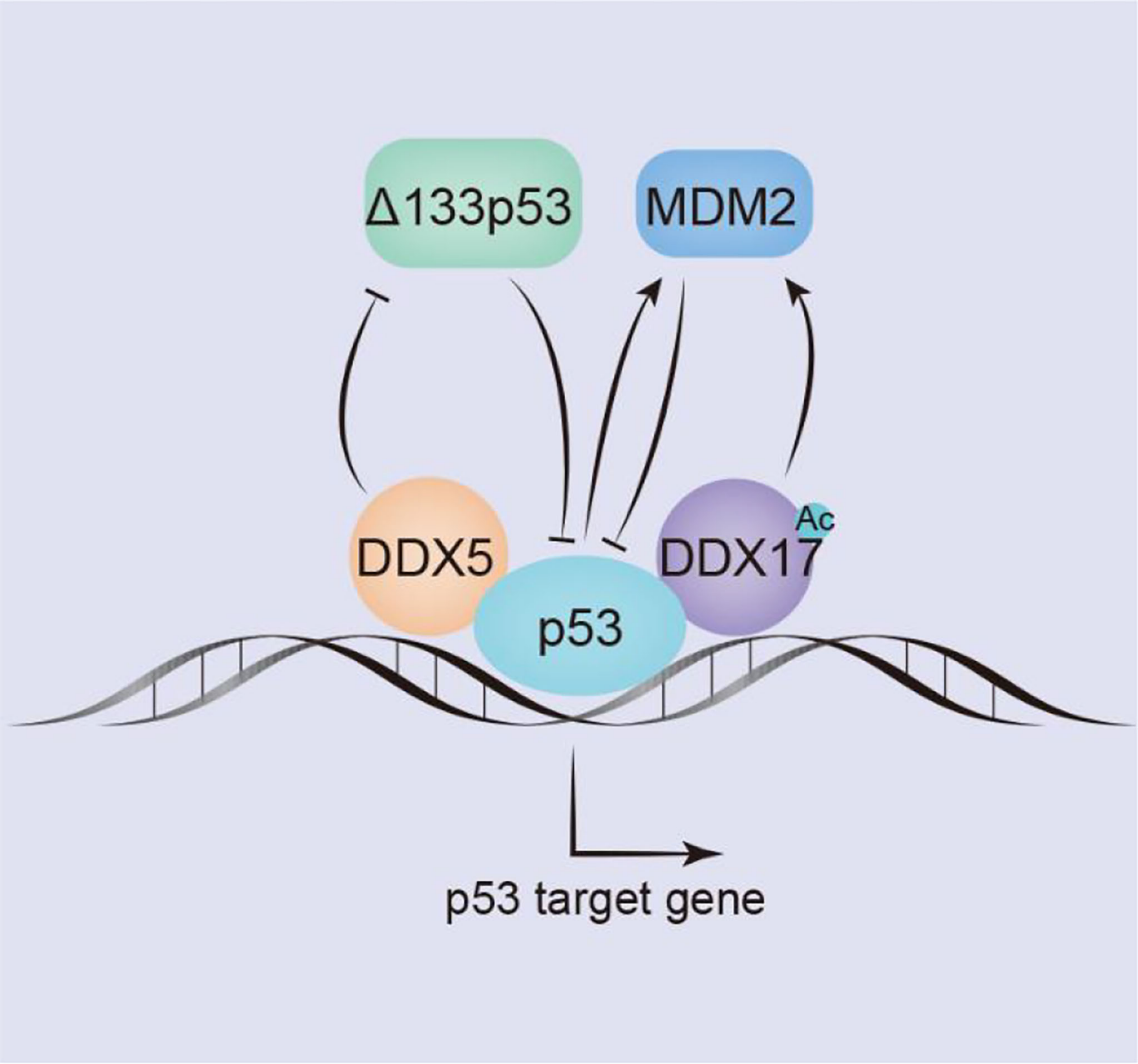
DDX5/DDX17 play an extremely important role in the p53 signaling pathway. DDX5/DDX17 are coreguulators of p53, and DDX5 selectively regulates p53 mediation of growth arrest or apoptosis; DDX5 positively regulates the expression of p53 by inhibiting Δ133p53. In contrast, DDX17 negatively regulates p53 expression by inducing Mdm2 expression.

P53 is one of the best-characterized tumor suppressors. When DNA damage is mild, p53 prevents DNA replication and growth stagnation, stalling transcription to allow cells time to repair the damage; when the sequence of damaged DNA cannot be restored, a cell undergoes apoptosis (121). In the luciferase reporter assay of H1299 (p53-null) cells (lung cancer cell line), DDX5/DDX17 interacted with p53 and acted as coactivators of p53, promoting the transcription of p53-targeted genes, but the co-activation ability of DDX17 is weaker than that of DDX5 (69). Knockdown experiments with short interfering RNA (siRNA) in MCF-2 cells (breast cancer cell line) have shown that DDX5 plays an important role in inducing the transcriptional activity of p53 in response to DNA damage but that knocking down DDX17 expression has no significant effect (69), suggesting that the effect on the p53 DNA damage response is specific to DDX5. In MCF-2 cells and U2OS cells (osteosarcoma cell line), DDX5 is recruited to the promoters of several p53-responsive genes, including those of the cell cycle arrest gene p21^WAF1^ and the proapoptotic genes Bax and PUMA; however, DDX5 appears to be required for selective p21^WAF1^ induction but not for proapoptotic genes (70). Therefore, during tumor treatment, the status of p53 and DDX5 can be assessed to evaluate the response to radiotherapy or chemotherapy and to select better cancer treatment strategies for patients (**Figure 3**).

P53 subtype–Δ133p53 is a negative regulator of full-length p53, which is overexpressed in many tumors. In breast cancers, DDX5 negatively regulates Δ133p53 production. The Δ133p53 subtype is not produced by alternative splicing; in contrast, it is produced by the transcription of an internal promoter in intron 4 of the p53 gene (71). Mdm2 inhibits the function of p53 by binding to the trans domain of p53 or by functioning as an E3 ubiquitin ligase to degrade p53 through the ubiquitin pathway; through both mechanisms, Mdm2 negatively regulates p53 level. However, p53 can act as a transcription factor to stimulate Mdm2 activation. In addition, Mdm2 transcription is regulated by the transcription factors AP1 and ETS. In colon and breast cancer cells, DDX17 activates the Mdm2 promoter and induces its transcription in a p53-dependent and p53-independent manner. The 63 amino acids in the N-terminus of DDX17 bind with the acetyltransferase p300/CBP and the related P/CAF protein to synergistically stimulate Mdm2 gene transcription. However, DDX17 may not directly contact the transcription factors AP1 or ETS but may indirectly act through CBP/p300 and/or P/CAF (86). In breast tumors, the acetylation of DDX17 enhances p53-dependent MDM2 promoter activation, stimulates MDM2 transcription, reduces the p53 level by promoting the p53-MDM2 negative feedback loop, and promotes carcinogenesis (117) (**Figure 3**).

#### 5.1.2 DDX5/DDX17 and β-catenin

β-catenin signaling is closely related to the tumorigenesis and proliferation of breast cancer, esophageal cancer, colon/colorectal cancer and non-small cell lung cancer (122). Platelet-derived growth factor (PDGF) activates cAbl to phosphorylate Y593 in DDX5. Phosphorylated DDX5 blocks the phosphorylation of β-catenin by GSK-3β and displaces Axin in the β-catenin complex to promote the nuclear translocation of β-catenin (115); moreover, DDX5 acts as a coactivator of β-catenin to promote the transcription of β-catenin target genes (such as *c-Jun*, *c-Myc*, *RelA*, and *cyclin D1*), which directly promote NSCLC and colon cancer cells proliferation (27, 123). In addition, β-catenin and transcription factor 4 (TCF4) can bind to the promoter of the DDX5 gene to stimulate the expression of DDX5, which in turn promotes the transcription of the β-catenin-dependent TCF4 gene, forming a positive feedback loop to promote breast cancer progression (124, 125). Hepatoma-derived growth factor (HDGF) interacts with DDX5 to induce β-catenin expression and stimulate its nuclear translocation by activating the PI3K/AKT signaling pathway to promote carcinogenesis of endometrial cancer (6). In addition, in colon cancer, DDX5, β-catenin and NF-κB synergistically promote AKT gene transcription, which leads to the phosphorylation of the tumor suppressor FOXO3a and excludes it from the nucleus, causing it to be degraded in the cytoplasm. Phosphorylation of FOXO3a reduces the expression of tumor suppressor p27kip1 and increases the expression of vascular endothelial growth factor (VEGF) and Cyclin D1, promoting the development of many types of tumors (126).

**Figure 4:**
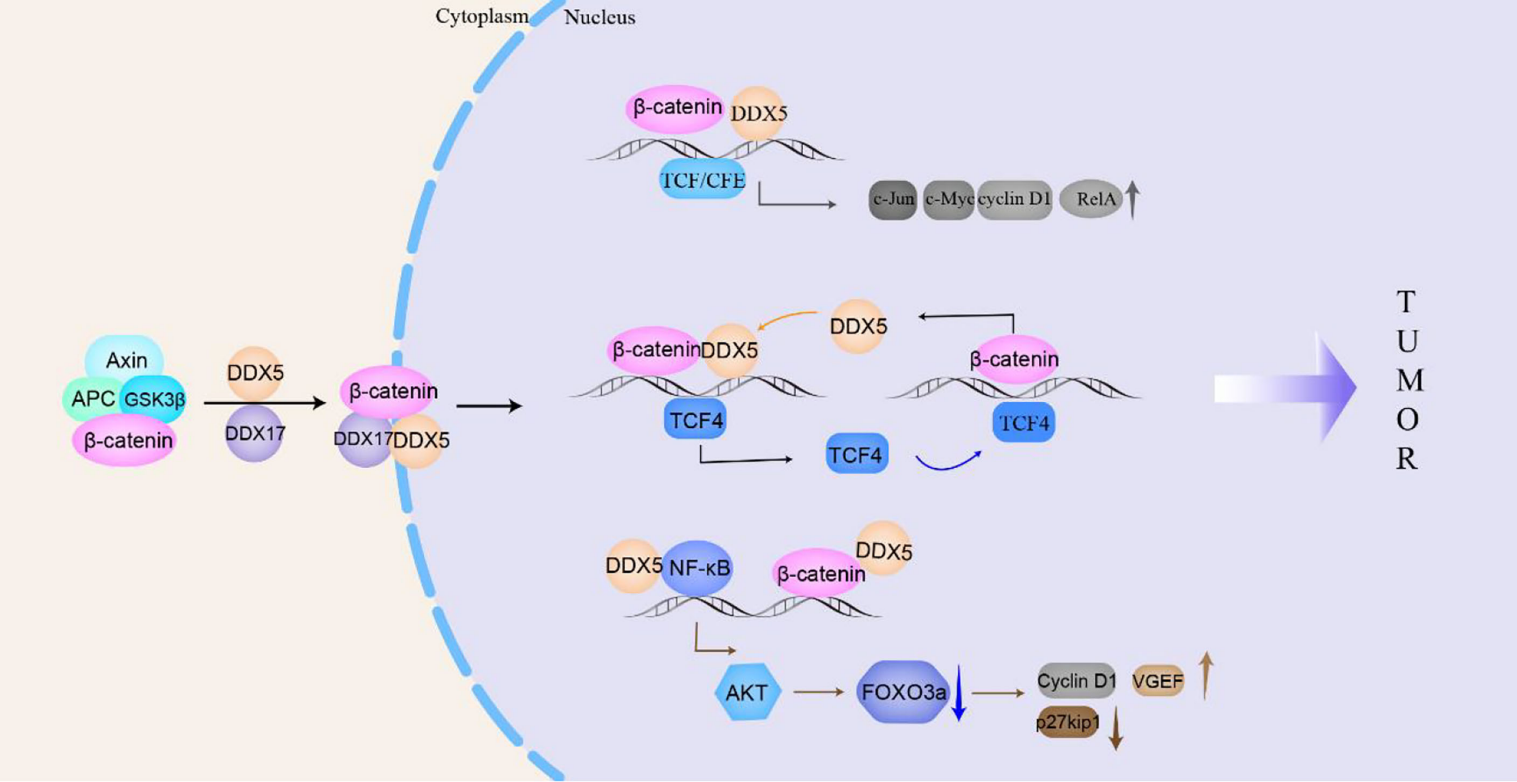
DDX5/DDX17 participate in the β-catenin signaling pathway to promote tumorigenesis and tumor progression. In the cytoplasm, DDX5/DDX17 dissociate β-catenin from the complex and transport it to the nucleus to promote the transcription of β-catenin target genes (*c-Jun*, *c-Myc*, *cyclin D1*, *TCF4*, *AKT*, etc.), which then participate in tumorigenesis and tumor progression.

Similar to DDX5, DDX17 can interact with cytoplasmic β-catenin to promote the dissociation of β-catenin from the E-cadherin/β-catenin complex. DDX17 may act as a molecular chaperone that transports β-catenin to the nucleus, increasing the accumulation of β-catenin in the nucleus and then increasing the transcription of β-catenin target genes (14). Gefitinib is used to treat NSCLC with EGFR mutations, but patients slowly acquire resistance to gefitinib. Compared with NSCLC cells that are sensitive to gefitinib, DDX17 expression in gefitinib-resistant cells is increased, and the Wnt/β-catenin signaling pathway is activated to ultimately render NSCLC cells resistant to gefitinib (14). In general, in the cytoplasm, DDX5/DDX17 promotes the dissociation of β-catenin from the complex and transports it to the nucleus to participate in the transcription of target genes, such as *c-Jun*, c*-Myc*, *cyclin D1*, *TCF4*, and *AKT*, then to promote the malignant progression of drug-resistant NSCLC, breast cancer, colon cancer, colorectal cancer, etc. (**Figure 4**).

#### 5.1.3 DDX5/DDX17, estrogen receptor, and androgen receptor

ERα and AR are members of the nuclear steroid hormone receptor family and play important roles in the development of breast cancer and prostate cancer, respectively (127, 128). In breast cancer, DDX5/DDX17, auxiliary activators of ERα, are recruited to the ERα-responsive promoter to promote gene transcription. However, only DDX17, not DDX5, is necessary in this process. In ERα-positive breast cancer, DDX5 expression positively correlates with the expression of the poor prognostic marker Her-2, while DDX17 expression correlates with a good prognosis and negatively correlates with the expression of Her-2 (68). Sox2-reactive breast cancer is more tumorigenic than nonreactive breast cancer. However, Alqahtani et al. found that in ERα-positive breast cancers that were responsive to Sox2, DDX17 was a transcriptional coactivator of Sox2 and promoted the transcription of Sox2 target genes to promote the malignant development of breast cancer (16). In prostate cancer, DDX5 is abnormally highly expressed. Studies have found that DDX5 was a transcriptional coactivator of AR. DDX5 interacts with AR and is recruited to the promoter region of androgen-responsive prostate-specific antigen genes to regulate gene expression. The phosphorylation of Y593 on DDX5 by c-Abl enhances the transcriptional coactivation of AR (5). In addition, the lncRNA CCAT1 serves as a scaffold for the DDX5 and AR transcription complex to promote the expression of AR-regulated genes, thereby promoting the progression of castration-resistant prostate cancer (73) (**Figure 1G**).

DDX5 and DDX17 are the main regulators of the estrogen and androgen signaling pathways (21). On the one hand, DDX5 and DDX17 control the splicing of several key regulators that can modulate the activity of ER and AR, acting upstream of ER and AR. On the other hand, DDX5 and DDX17 modulate the transcription and splicing of a large number of steroid hormone target genes, acting downstream of ER and AR.

#### 5.1.4 DDX5/DDX17 and NF-κB

**Figure 5:**
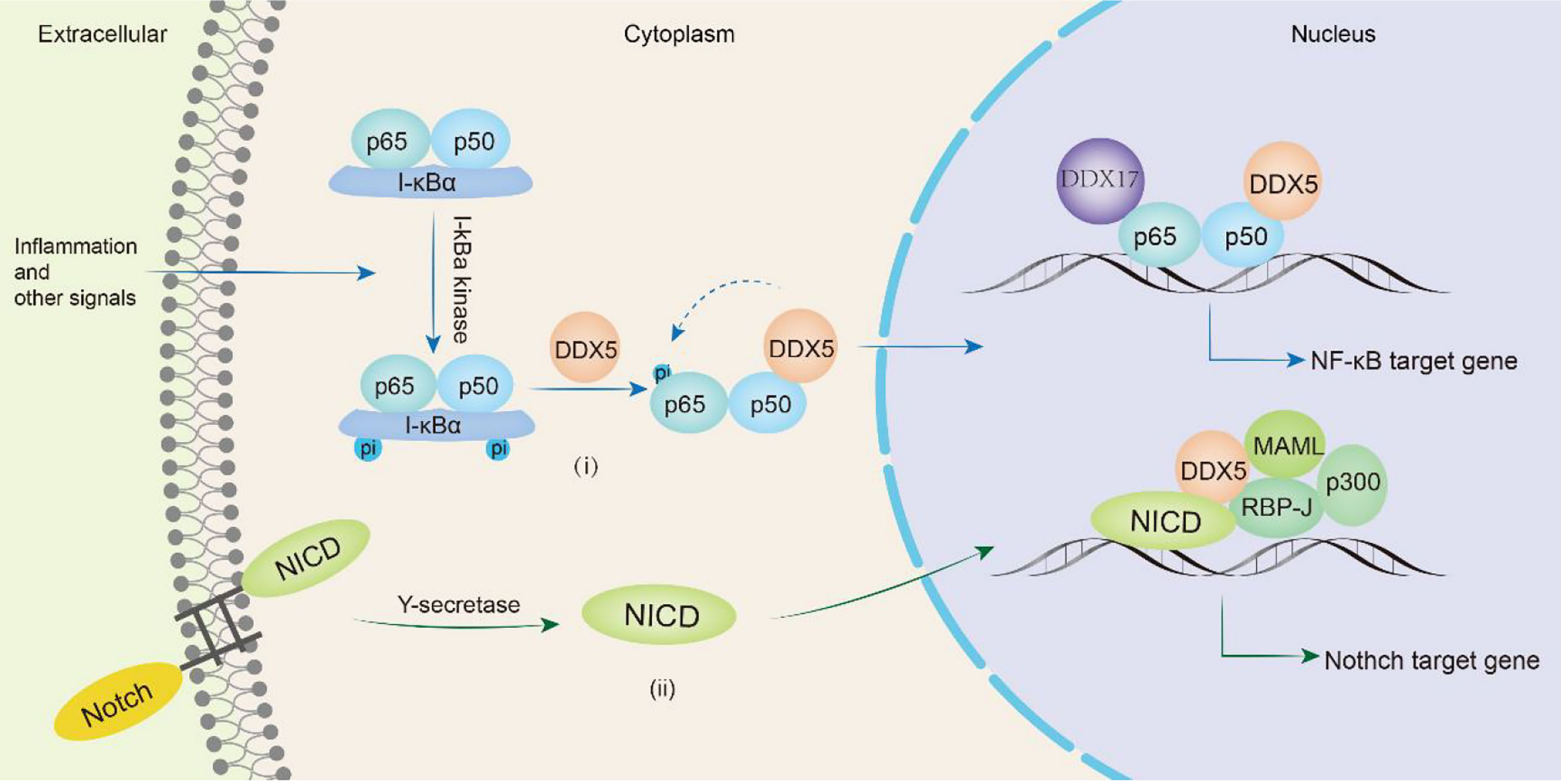
DDX5/DDX17 are involved in tumor progression through NF-κB and Notch signaling pathways. In the NF-κB signaling pathway(i), DDX5 promotes the release of the inhibitory subunit IκBα, induces nuclear translocation of the p50 subunit, and in directly phosphorylates the p65 subunit, thereby promoting the transcription of NF-κB target genes. DDX17 is involved in alternative splicing of p65 subunit target genes such as CD44. In the Notch signaling pathway(ii), DDX5 binds MAML1 and is recruited to the Notch We would like to delete the entire glossary section. Please see below. transcription complex to promote the transcription of target genes.

In mammals, there are five proteins in the NF-κB family, RelA (p65), RelB, c-Rel, NF-κB1 (p50) and NF-κB2 (p52), which can form homologs or heterodimers to regulate gene transcription. NF-κB can respond to external stimuli, including radiation and viral infection, and participate in cellular inflammatory and immune responses (129). Abnormal NF-κB expression can lead to cancers and autoimmune diseases. In glioma patients, DDX5 expression is significantly associated with poorer overall survival. Wang et al. found that DDX5 induced glioma tumor growth by regulating NF-κB p50 nuclear accumulation and transcriptional activity. The N-terminus of DDX5 binds to the p50 subunit of NF-κB to promote the release of the inhibitory subunit IκBα, inducing the accumulation and transcription of p50 in the nucleus and promoting the transcription of NF-κB p50 target genes (130). DDX5 knockdown does not influence the nuclear translocation of the NF-κB p65 subunit but selectively inhibits the phosphorylation of Ser311 in the p65 subunit, thus selectively inhibiting the expression of the antiapoptotic protein Bcl-2, which makes cells susceptible to apoptosis. However, DDX5 does not directly bind to the p65 subunit and may indirectly regulate the NF-κB system through a regulatory complex (131). The lncRNA PRADX binds to the enhancer of zeste homolog 2 (EZH2) protein, recruits the PRC2/DDX5 complex, increases the abundance of H3K27me3 on the UBXN1 promoter, inhibits the expression of UBXN1, then promotes the activity of NF-κB, thus promoting the development of glioblastoma and colon adenocarcinoma (106). In addition, DDX5 and β-catenin jointly positively regulate the expression of NF-κB target genes to promote colon carcinogenesis (123). Adult T-cell leukemia/lymphoma (ATLL) is a malignant T-cell monoclonal proliferative disease caused by human T-cell leukemia virus type I (HTLV-1). The Tax protein encoded by HTLV-1 triggers the NF-κB signaling pathway to induce malignant cell proliferation. After NF-κB signaling is activated, RELA binds to GC-rich exon genes, recruits DDX17, and utilizes the helicase activity of DDX17 to regulate CD44 alternative splicing to promote the progression of ATLL (34). Therefore, DDX5 or DDX17 is involved in the progression of various cancers (glioma, colon cancer, glioblastoma, colon adenocarcinoma, adult T-cell leukemia/lymphoma) by participating in the NF-κB signaling pathway (**Figure 5i**).

#### 5.1.5 DDX5 and Notch

The Notch signaling pathway is a highly conserved intercellular communication pathway that regulates normal cell development and tissue homeostasis. It is an important target in cancers including breast, lung, pancreatic, and brain cancers, melanoma and T-cell acute lymphoblastic leukemia (T-ALL) (132). The signaling molecule binds to the Notch receptor and releases the Notch intracellular domain (NICD), which is translocated to the nucleus where it forms a complex with RBP-J. The NICD/RBP-J complex binds to MAML, DDX5 and SRA and then coactivates the transcription of Notch target genes *(preTCRα*, *Hes1* and *CD25*) (78). During this process, DDX5 binds to MAML1 and is recruited to the Notch transcriptional activation complex, which is located on the Notch response promoter HES1 and regulates Notch-induced transcription (133). Compared with that in the normal control group, DDX5 expression was upregulated in the bone marrow and peripheral blood samples of human T-ALL patients. Knocking down DDX5 reduces the expression of the Notch signaling gene in leukemia cells and inhibits the proliferation of leukemia cells, resulting in a reduction in the growth of leukemia xenotransplants and promoting cell apoptosis (133). Thus, DDX5 overexpression plays a key role in the pathogenesis of T-ALL mediated by Notch (**Figure 5ii**).

#### 5.1.6 DDX17 and YAP

The Hippo signaling pathway is sensitive to cell connections and density, which leads to differences in the subcellular localization of the transcription coactivator Yes-associated protein (YAP) (134). In tumor cells, that is, at low cell density, Hippo signaling is inhibited, and YAP is located in the nucleus, where it serves as a transcription-assisted activator to promote cell proliferation. The WW1 domain of YAP binds DDX17 and prevents DDX17 from acting on microprocessors, which inhibits miRNA biosynthesis and leads to the downregulation of miRNA density in cells. When the cell density is relatively high, YAP is phosphorylated and isolated in the cytoplasm by E-cadherin and α-catenin, and YAP is excluded from the nucleus and cannot be activated, enabling DDX17 to act on microprocessors and participate in the biosynthesis of miRNA (135). Global downregulation of miRNAs is usually observed in cancer; therefore, failure of the Hippo signaling pathway may result in widespread inhibition of miRNA generation in tumor cells to promote cancer development.

**Figure 6:**
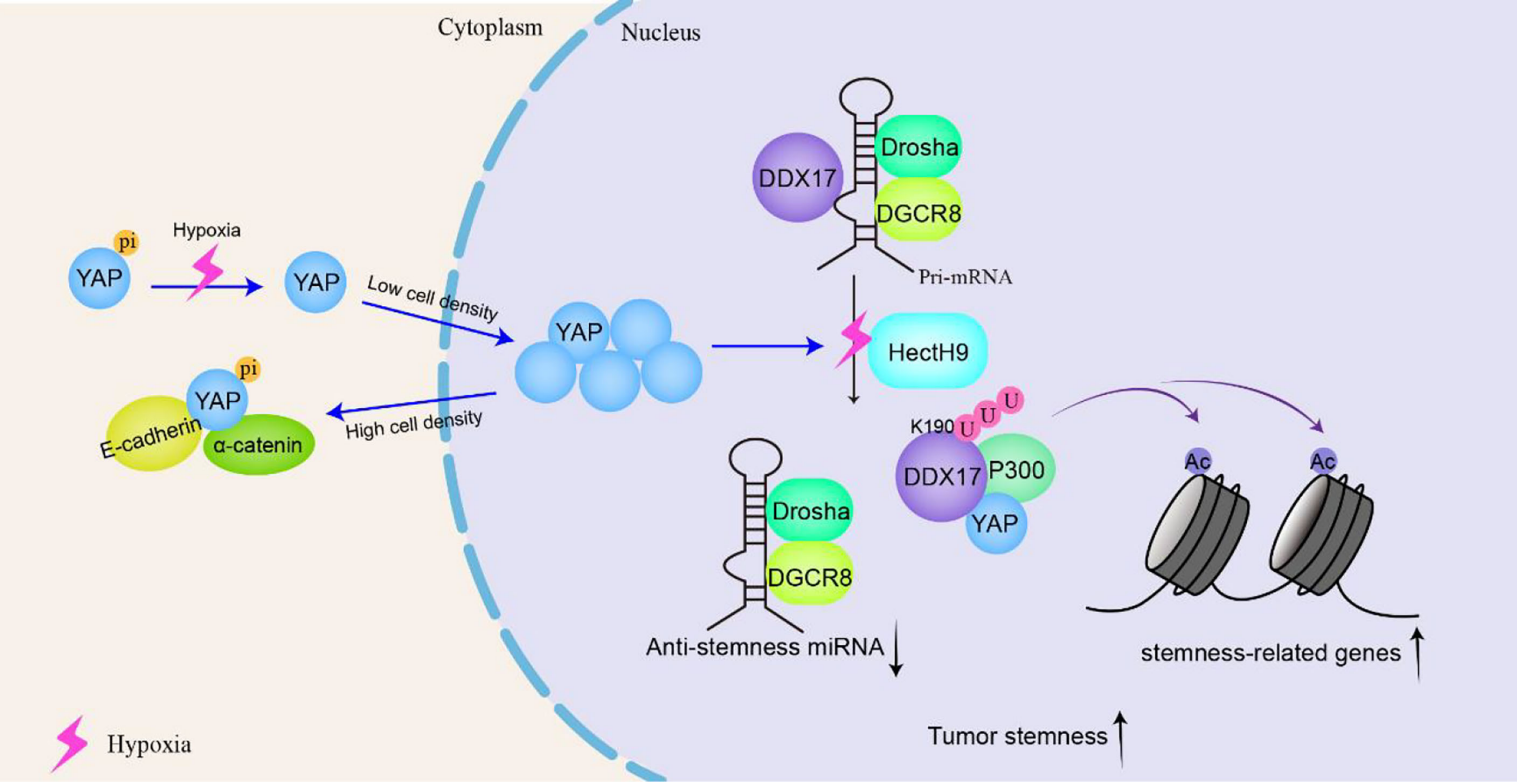
DDX17 participates in the YAP signaling pathway to increase the stemness of cancer stem-like cells and to promote tumorigenesis and tumor progression. In tumor cells, that is, at low density, YAP is located in the nucleus and serves as a transcription-assisted activator to promote cell proliferation. Under hypoxic conditions, K190 on DDX17 undergoes K63 ubiquitination by the E3 ligase HectH9. YAP binds to and isolates ubiquitinated DDX17, dissociating it from the Drosha-DGCR8 complex to block the biosynthesis of antitumor stem microRNAs (miRNAs). Meanwhile, ubiquitinated DDX17 forms a YAP-DDX17-p300 complex, leading to the acetylation of histone 3 lysine 56 (H3K56) to activate cancer-related gene transcription, resulting in increased stemness of cancer stem-like cells.

Cancer stem cells (CSCs) can self-renew and differentiate into mature tumor cells in tumors. The stem cell-like characteristics of CSCs are related to higher tumorigenicity, cancer recurrence and metastasis in patients. In locally advanced solid tumors, hypoxia is an important microenvironmental factor that leads to malignant progression by increasing tumor cell stemness. Under hypoxic conditions, K190 in DDX17 is induced to K63 ubiquitination under the action of the E3 ligase HectH9, and YAP is subject to dephosphorylation through the SIAH2-LATS signaling pathway. The dephosphorylation of YAP promotes its nuclear translocation efficiency and increases the YAP concentration in the nucleus. YAP binds to and isolates ubiquitinated DDX17, dissociating it from the Drosha-DGCR8 complex and thus blocking the biosynthesis of antitumor stem miRNA. In addition, the K63 polyubiquitination of DDX17 increases the DDX17 interaction with the ubiquitin-binding protein p300, forming a YAP-DDX17-p300 complex. Because P300 is a histone acetyltransferase, the complex leads to the acetylation of histone 3 lysine 56 (H3K56) near tumor-related genes and subsequently activates the expression of these genes (e.g., BMI1, SOX2, and OCT4) (118). Therefore, ubiquitinated DDX17 coordinates the regulation of miRNA biogenesis and histone modification, which is the basis of many CSC-like characteristics (**Figure 6**).

In addition to interacting with p53, Wnt/β-catenin, Notch, ER, AR, and NF-κB, DDX5 or DDX17 engage in crosstalk with mTOR (136, 137), Smad3 (138), STAT3 (77), Akt (139), c-Myc (140), TGF-β1 (141), etc. DDX5/DDX17 interact with an overwhelming number of key tumor factors, and therefore, the importance of DDX5/DDX17 cannot be overemphasized. When DDX5/DDX17 expression is disrupted, the body is at great risk of developing cancer.

### 5.2 Other tumor regulatory signaling pathways

DDX5 or DDX17 also participate in other tumor regulatory signaling pathways, such as DNA repair, oxidative stress, autophagy, and energy metabolism.

#### 5.2.1 Affecting DNA repair

DDX5 can affect DNA repair. For example, Yuan et al. found that DDX5 can promote the development of thyroid cancer by stimulating DNA repair signaling. The lncRNA SLC26A4-AS1 acts as a scaffold linking DDX5 and the E3 ligase to promote DDX5 degradation through the ubiquitin–proteasome pathway. DDX5 is a coactivator of E2F1 (80). In human thyroid cancer cells, SLC26A4-AS1 silencing enhances the interaction between DDX5 and the transcription factor E2F1; then, the DDX5-E2F1 complex binds to the MRN gene promoter and thus stimulates the MRN/ATM-dependent DNA DSB signaling cascade and thyroid cancer metastasis (97). However, Zhao et al. found that DDX5 downregulation promoted the resistance of osteosarcoma to camptothecin by inhibiting DNA repair. Although DDX5 expression in osteosarcoma cells was higher than that in normal bone cells, its expression level was generally lower. Camptothecin induced the degradation of DDX5, which bound to the DNA repair protein NONO. The reduction in DDX5 promoted the release of NONO, which participated in DNA repair in human osteosarcoma cells, thereby downregulating DDX5 induced the resistance of osteosarcoma cells to camptothecin (96). The two experiments appear to have opposite conclusions, but they occur in different cancer tissues; moreover, the effects of DDX5 in different environments can be quite different. However, these two experiments also show that DDX5 expression levels that are too high or too low will increase the risk of cancer migration and lesions. DDX17 is also involved in DNA repair. In amyotrophic lateral sclerosis (ALS), DDX17 upregulation helps repair DNA damage caused by FUS and inhibits FUS-induced neurotoxicity. However, there is no report on how DDX17 involved in DNA repair affects cancer. This question will be an interesting and potential topic to investigate in the future (142).

#### 5.2.2 Inhibiting ROS production

DDX5 can inhibit the production of reactive oxygen species (ROS). Acute myeloid leukemia (AML) patients have a complex karyotype and distinct abnormal expression of DEAD-box family proteins. Inhibiting DDX5 expression promotes the production of ROS, thereby inhibiting the proliferation of AML cells and inducing their apoptosis, but DDX5 inhibition is not toxic to normal bone marrow cells (143). Wu et al. (144) prepared a fully human monoclonal antibody targeting DDX5 and named it 2F5, which selectively inhibited the proliferation of acute promyelocytic leukemia (APL) cells and was nontoxic to normal neutrophils and tissues. 2F5 inhibited APL cell proliferation and promoted their differentiation by targeting DDX5 to induce ROS generation, suggesting a new and effective method for the treatment of refractory/relapsed APL. However, Wu et al. also found that 2F5 exerted no effect on the proliferation of T-ALL cell lines (144). The reasons for these different outcomes may be explained by variance in basal DDX5 expression in different leukemia cell lines; for example, DDX5 expression in APL cell lines is significantly higher than that in T-ALL cell lines. Thus, DDX5 expression determines the susceptibility of different leukemia subtypes to 2F5.

#### 5.2.3 Regulating cell autophagy

DDX5 can regulate cancer development by affecting cell autophagy. In liver cancer, DDX5 overexpression significantly reduces tumorigenesis, and patients with low DDX5 expression may have a poorer prognosis after treatment. DDX5 overexpression induces autophagy in liver cancer cells. DDX5 promotes p62 degradation by interacting with the p62 TBS domain and then interrupts p62 binding to tumor necrosis factor receptor-associated factor 6 (TRAF6) and thus reduces the K63 polyubiquitination of mTOR to inhibit mTOR signaling transduction (2). However, in esophageal squamous cell carcinoma, DDX5 increases endoplasmic reticulum stress and reduces autophagic flux to promote cancer proliferation and metastasis (145). In glioma, DDX17 promotes glioma cell invasion by inhibiting autophagy. In A172 cells and T98G cells (glioma cell lines), DDX17 controls the biosynthesis of miR-34-5p and miR-5195-3p, respectively; both of these miRNAs target Beclin1 to inhibit autophagy and promote the migration and invasion of glioma cells (146). This study indicates that DDX17 is a negative prognostic factor.

#### 5.2.4 Promoting respiratory metabolism

DDX5 supports the energy supply in cancer cells by promoting respiratory function. DDX5 is overexpressed in small cell lung cancer (SCLC) cell lines. DDX5 deletion decreases the expression of oxidative phosphorylation-related genes in the drug-resistant H69AR SCLC cell line, reducing oxygen consumption, causing mitochondrial dysfunction, and inhibiting cell growth. Succinic acid is an intermediate product of the tricarboxylic acid cycle and a direct electron donor of mitochondrial complex II, and its upregulation is positively correlated with chemotherapeutic resistance. Studies have shown that the lack of DDX5 reduced succinate in cancer cells (147). These findings indicate that the carcinogenic effect of DDX5 is at least partially manifested as an upregulation of mitochondrial respiration and support for the energy requirements of cancer cells.

## 6 sTherapeutic prospects

High DDX5 expression increases the recurrence rates of breast cancer (4), hepatocellular carcinoma (148), glioma (17), and squamous cell carcinoma (145), shortening the clinical survival time. Similarly, high DDX17 expression leads to a worsened prognosis for patients with glioma (17), gastric cancer (149), pancreatic cancer (150), colon cancer (151), paclitaxel-resistant ovarian cancer (152), and other cancers. For example, DDX5/DDX17 expression significantly correlates with the WHO grade and histological type of glioma patients. Patients with high DDX5/DDX17 expression present with higher grade malignancy and shorter clinical survival times (17). Therefore, DDX5/DDX17 can be used as clinical biomarkers for a cancer diagnosis and for prognosis prediction.

Moreover, many anticancer drugs inhibit cancer by affecting DDX5/DDX17 activity. For instance, simvastatin inhibits renal cell carcinoma cell proliferation by reducing DDX5 expression (153); resveratrol inhibits prostate cancer growth by promoting DDX5 degradation (136); the tumor suppressor DRD2 inhibits breast cancer by downregulating DDX5 expression (154); 2F5, the DDX5-targeting fully human monoclonal autoantibody, selectively inhibited the proliferation of acute promyelocytic leukemia cells (144). and endoxifen and fulvestrant, which are endocrine therapy drugs, inhibit breast cancer by downregulating DDX5/DDX17 expression (155). Recently, supinoxin (RX-5902), a small-molecule inhibitor of DDX5, has been developed for cancer therapy and is currently in clinical trials with metastatic triple-negative breast cancer patients (6, 156–158). Therefore, the successful development of DDX5-targeting drugs further demonstrates the great potential of using DDX5 in the field of tumor therapy (**Table 3**).

**Table 3:** Anticancer drugs target DDX5/17 to inhibit tumor progression.

Anticancer drugs	Targeting DDX	Cancer types	References
Simvastatin	DDX5	Renal cell carcinoma	(153)
Resveratrol	DDX5	Prostate cancer	(136)
DRD2	DDX5	Breast cancer	(154)
2F5	DDX5	Acute promyelocytic leukemia	(144)
Endoxifen/Fulvestrant	DDX5/17	Breast cancer	(155)
RX-5902	DDX5	Triple-negative breast cancer	(156–158)

Therefore, DDX5 and DDX17 both show great potential in the prediction, diagnosis and treatment of many types of tumors. First, DDX5/DDX17 can be used as biomarkers for predicting cancer. When DDX5 and DDX17 expression is abnormal in the physical examination, it indicates that the patient’s health status is poor, with increased the risk of DDX5-/DDX17-associated cancer. Second, DDX5 and DDX17 can be used as clinical predictive molecules for predicting cancer recurrence and prognosis. In most cancers, when DDX5/DDX17 expression levels are elevated, the risk of malignancy is higher, and clinical survival time is shorter. Third, regulating the posttranslational modification of DDX5/DDX17 can change their functions and targets, improving the tumor response to anticancer drugs and reducing the drug resistance of malignant tumors. Fourth, it is very important to develop DDX5/DDX17 inhibitors and targeted therapy methods for tumor treatment. The successful development of RX-5902 has proven that this approach is feasible.

## 7 Summary

DDX5/DDX17 are involved in almost all RNA metabolism processes, such as RNA unwinding, secondary structure rearrangement, mRNA selective splicing, miRNA and rRNA biosynthesis, sense-mediated mRNA degradation, and interaction with transcription factors and lncRNAs. DDX5/DDX17 are involved in different posttranslational modifications. Even the same posttranslational modifications at different modification sites can lead to different biological effects. In addition, DDX5/DDX17 interact with important tumor signaling molecules, and DDX5 is involved in DNA repair, oxidative stress, autophagy, and energy metabolism. Therefore, DDX5/DDX17 exhibit a wide range of biological functions. Once DDX5/DDX17 expression or DDX5/DDX17-related posttranslational modification is dysregulated, the cellular signaling network collapses or becomes abnormal, which leads to the acquisition of many pathological states, including those related to tumorigenesis and tumor development.

Interestingly, some similar studies on DDX5/DDX17 often yielded opposite results or conclusions, which may have been due to the functions of these proteins being highly dependent on the environment, such as cell or tissue type, subcellular distribution, cell density, pathological conditions (such as hypoxia), etc. Therefore, many factors may have contributed to the conflicting results of certain experiments.

There are still some problems with the current research. DDX17 can be translated into two proteins, p72 and p82; however, p82 has rarely been studied. In most studies, only p72 mRNA or both p72 and p82 mRNA was knocked down in the experiments. Therefore, little is known about p82 and the functional differences between p72 and p82, and their potential differences need to be further explored.

In summary, DDX5 and DDX17 regulate tumorigenesis and tumor progression by participating in RNA metabolism, regulating posttranslational modifications, acting on tumor-related signaling pathways, etc (**Table 4**). However, most recent DDX5/DDX17 research has been limited to basic research, and additional research needs to be performed in the broader clinical field.

**Table 4:** Expression and mechanism of DDX5/17 in different cancers.

Cancer type	DDX5/17	DDX5/17expression	mechanisms/signaling	References
Breast cancer	DDX5/17	↑	DDX5/DDX17 are auxiliary activators of ERα DDX17 is a transcriptional coactivator of Sox2 and MDM2 DDX5 participates in β-catenin signaling pathway	(16, 68, 117)
Non-small-cell lung cancer	DDX5/DDX17	↑	Participate in β-catenin signaling pathway	(23)
Adult T-cell leukemia/lymphoma	DDX17/DDX5	↑	DDX17 participates in NF-κB signaling pathway and alternative splicing of CD44 DDX5 participates in Notch signaling pathway	(34, 78, 133)
Glioma tumor	DDX5/DDX17	↑	DDX5 participates in NF-κB signaling pathway; DDX17 inhibits glioma cell autophagy	(130, 146)
Prostate cancer	DDX5	↑	DDX5 is a transcriptional coactivator of AR	(73)
Colon cancer	DDX5	↑	Participate in β-catenin and NF-κB signaling pathway	(123)
colorectal carcinoma	DDX5	↑	Participate in β-catenin signaling pathway	(125)
Endometrial Cancer	DDX5	↑	Participate in β-catenin signaling pathway	(6)
Colon cancer	DDX5	↑	Participate in β-catenin signaling pathway	(126)
glioblastoma	DDX5	↑	Participate in NF-κB signaling pathway	(106)
colon adenocarcinoma	DDX5	↑	Participate in NF-κB signaling pathway	(106)
Thyroid cancer	DDX5	↑	Stimulate DNA DSB signaling cascade	(97)
Acute promyelocytic leukemia	DDX5	↑	Inhibiting ROS production	(144)
Esophageal squamous cell carcinoma	DDX5	↑	Regulating cell autophagy	(145)
Small cell lung cancer	DDX5	↑	Promote respiratory metabolism	(147)
Liver cancer	DDX5	↓	Regulating cell autophagy	(2)
Pancreatic ductal adenocarcinoma	DDX5	↓	Low expression of DDX5 is associated with poor prognosis in patients	(3)
pancreatic ductal adenocarcinoma	DDX17	↑	Participate in the alternative splicing of Caspase 9, mH2A1	(37)
hepatocellular carcinoma	DDX17	↑	DDX17 is a cosuppressor of Klf4 and promote the generation of the PXN-AS1-IR3 transcript	(18, 36)

## Author contributions

KX is responsible for conceptualization, writing original draft, and figure Preparation. SS, MY, JC, YY, and WL are responsible for figure Preparation. XH, LD, BC, WT, ML and JL are responsible for writing-reviewing. TS is responsible for supervision and writing-reviewing and editing. All authors contributed to the article and approved the submitted version.

## Funding

This study was supported by grants from the National Natural Science Foundation of China (grant nos. 81770228, 81770858, 81600618, 82073264, 82170890 and 81470427), the Beijing Natural Science Foundation (7142142), the National Key R&D Program of China (2018YFC2000100), National High Level Hospital Clinical Research Funding (BJ-2021-199), and the Chinese Academy of Medical Sciences (CAMS) Innovation Fund for Medical Sciences (no. 2021-I2M-1-050).

## Acknowledgements

We would like to thank the Key Laboratory of Geriatrics Beijing Hospital and Beijing Institute of Geriatrics for their support of this review.

## Conflict of interest

The authors declare that the research was conducted in the absence of any commercial or financial relationships that could be construed as a potential conflict of interest.

## Publisher’s note

All claims expressed in this article are solely those of the authors and do not necessarily represent those of their affiliated organizations, or those of the publisher, the editors and the reviewers. Any product that may be evaluated in this article, or claim that may be made by its manufacturer, is not guaranteed or endorsed by the publisher.
