# Corrigendum: DDX5 and DDX17—multifaceted proteins in the regulation of tumorigenesis and tumor progression

**DOI:** 10.3389/fonc.2023.1204712

**Published:** 2023-06-02

**Authors:** Kun Xu, Shenghui Sun, Mingjing Yan, Ju Cui, Yao Yang, Wenlin Li, Xiuqing Huang, Lin Dou, Beidong Chen, Weiqing Tang, Ming Lan, Jian Li, Tao Shen

**Affiliations:** ^1^ The Key Laboratory of Geriatrics, Beijing Institute of Geriatrics, Institute of Geriatric Medicine, Chinese Academy of Medical Sciences, Beijing Hospital/National Center of Gerontology of National Health Commission, Beijing, China; ^2^ Peking University Fifth School of Clinical Medicine, Beijing, China

**Keywords:** DDX5, DDX17, RNA transcription regulation, posttranslational modification, tumorigenesis, tumor progression

In the published article, there was an error. The wrong number of amino acids.

A correction has been made to Structural features of DDX5 and DDX17, [2]. This sentence previously stated:

“The transcription and translation of the DDX5 gene produces a stable protein with a molecular weight of 68 kDa, also known as p68, comprising 621 amino acids.”

“these proteins are also known as p72 and p82, respectively, and comprise 729 and 731 amino acids.”

The corrected sentence appears below:

“The transcription and translation of the DDX5 gene produces a stable protein with a molecular weight of 68 kDa, also known as p68, comprising 614 amino acids.”

“these proteins are also known as p72 and p82, respectively, and comprise 650 and 729 amino acids.”

The authors apologize for this error and state that this does not change the scientific conclusions of the article in any way. The original article has been updated.

